# Medical Missionary Society’s Hospital at Canton

**Published:** 1860-11

**Authors:** 


MEDICAL MISSIONARY SOCIETY'S HOSPITAL AT CANTON.—We have received from our friend and collaborator, Dr. J. G. Kerr, a report of this hospital for the year 1859, and are happy to congratulate him upon the success which has attended his efforts as a medical missionary in the Celestial Empire. The institution is situated in the southern suburbs, opposite the middle of the walled city, and has recently received an appropriation of sixteen hundred dollars for enlarging its accommodations.

During 1859, thirteen thousand one hundred and eighty-six patients were prescribed for, and fifty-nine in-patients admitted. About seventy-five operations were performed, including seven for cataract, eleven for the removal of tumors, two for hydrocele, six for carious bone, three for urinary calculus, forty for entropion, two for pterygium, one for perineal section, and one for neuralgia of the inferior maxillary nerve. The subjects of the calculi, as well as of the other important operations, all recovered.

The most common diseases treated by Dr. Kerr were those of the eye, bronchitis, dropsy, intermittent fever, rheumatism, scrofula, ulcers, and affections of the skin. Large quantities of quinine have been introduced into the market and sold to the Chinese, who greatly appreciate its remedial value; and a number of steel trusses have been distributed, at cost price, to such as could afford to pay for them. The supply was not, however, equal to the demand, as hernia is a very common affection. A vaccine department has been established, and on this subject Dr. Kerr makes the following remarks:—

"Vaccination was introduced among the Chinese in this city by Dr. Pearson, about the year 1805. Owing to the persevering efforts of this gentleman, continued through many years, the inestimable blessings of this preventative are fully known to the Chinese, and his name is worthy of little less honor than that of the immortal Jenner, who first made it known to the world. Small-pox is almost an annual epidemic in China, and without the aid of government or any public institutions it is not strange that vaccination is neglected by multitudes in so vast a population. Those who practice vaccination are not able to preserve the virus, and they depend for fresh supplies brought from England. It was with the design of supplying these deficiencies that the department was established, and it is hoped that while gratuitous vaccination is offered to the poor, the hospital will be a source from which to supply virus at all times to all parts of the south of China. A tract on the subject has been prepared and printed, in which directions are given for preserving the scab and vaccinating from it."

